# Phenotypic, Genetic, and Virulence Characterization of *Tenacibaculum maritimum* Isolates Recovered from Salmonid Outbreaks in Chile

**DOI:** 10.3390/pathogens15070744

**Published:** 2026-07-15

**Authors:** Sara Valdes, José Miguel Saavedra, Eugenia Jerez, Elida Lebtun, Roxana Vargas, Pabla Barra, Jorge R. Toledo, Jaime Romero, Harold Oliva, Pedro Ilardi

**Affiliations:** 1 Farmacología en Aquacultura Veterinaria FAV S.A.L., Santiago 9020000, Chile; 2 Biotechnology and Biopharmaceutical Laboratory, Pathophysiology Department, School of Biological Sciences, Universidad de Concepción, Concepcion 4070386, Chile; 3 Laboratorio de Biotecnología de Alimentos, Instituto de Nutrición y Tecnología de los Alimentos (INTA), Universidad de Chile, El Líbano 5524, Santiago 7830489, Chile; 4 Cooperative Program for Aquaculture (Universidad de Chile, Universidad Católica del Norte, Pontificia Universidad Católica de Valparaíso), Universidad de Chile, El Líbano 5524, Santiago 7830489, Chile

**Keywords:** tenacibaculosis, salmon aquaculture, molecular genotyping, serotyping, Chilean isolates, *Genypterus chilensis*

## Abstract

*Tenacibaculum maritimum* is a major etiological agent of tenacibaculosis in marine fish and represents an increasing concern for Chilean salmon aquaculture; however, updated information on the phenotypic and molecular diversity of circulating isolates is limited. This study characterized 40 isolates recovered from Atlantic salmon (*Salmo salar*), rainbow trout (*Oncorhynchus mykiss*), and red cusk eel (*Genypterus chilensis*) obtained from outbreaks between 2020 and 2024. Isolates were analyzed using biochemical and phenotypic assays, multiplex PCR targeting the O-antigen gene cluster (O-AGC), REP-PCR-based genetic fingerprinting, and experimental bath challenges in Atlantic salmon. Phenotypic characterization revealed species-consistent traits but variable gelatin and starch hydrolysis and differences in seawater tolerance. O-AGC typing identified four molecular serotypes (1-0, 3-1, 3-2, and 4-0), with serotypes 1-0 and 3-2 detected for the first time in Chilean salmonids. Genetic fingerprinting distinguished previously described profiles and two novel REP patterns (REP6 and REP7), indicating additional genomic heterogeneity within dominant serotypes. Virulence assays showed cumulative mortality ranging from 0% to 100%, with serotype 3-1 isolates generally associated with higher mortality and serotype 4-0 displaying broad intra-serotype variability. These findings document substantial phenotypic, antigenic, and genetic diversity among Chilean *T. maritimum* isolates and provide epidemiologically relevant information for disease surveillance and vaccine design in salmon aquaculture.

## 1. Introduction

Tenacibaculosis is a severe bacterial disease affecting a wide range of marine fish species and represents a persistent challenge for aquaculture worldwide [1,2,3]. In salmonids, outbreaks have been reported in all major salmon-producing regions, including Tasmania [4], Canada [5], Norway [6], and the British Isles [7]. In Atlantic salmon (*Salmo salar*) and rainbow trout (*Oncorhynchus mykiss*), the disease is typically characterized by skin erosions, mouth lesions, fin necrosis, reduced feeding, lethargy, and impaired osmoregulation, which may predispose fish to secondary infections [8]. Mortality patterns vary according to fish developmental stage and environmental conditions, with smolts often exhibiting acute outbreaks.

Tenacibaculosis is caused by several species within the genus *Tenacibaculum*, members of the family Flavobacteriaceae [9]. Among them, *T. maritimum* remains one of the most frequently reported and economically relevant species associated with salmonid outbreaks worldwide. Although originally described in marine flatfish, its host range includes multiple farmed species across different geographic regions [2,10].

In Chile, tenacibaculosis has gained increasing epidemiological relevance during the marine grow-out phase of salmon farming. Since its inclusion in the national high-risk disease list (Exempt Resolution 2574, SUBPESCA, 2018 (https://www.subpesca.cl/portal/615/articles-101155_documento.pdf) (accessed on 20 February 2026)), systematic surveillance has documented a sustained rise in associated mortality. According to official reports, mortality attributed to tenacibaculosis increased from 4.3% in 2018 to 29.4% in 2024 in Atlantic salmon grow-out centers (SERNAPESCA, 2024 (https://www.sernapesca.cl/informes-y-datos/resultados-de-gestion/ (accessed on 20 February 2026)). In recent years, multiple *Tenacibaculum* species have been identified in Chilean salmonid production systems, including *T. dicentrarchi* [11], *T. maritimum* [12,13,14]; *T. finnmarkense* [15], *T. piscium* [16], *T. ovolyticum* [17], *T. bernardetii* [18], and more recently *T. salmonis* [19].

Previous studies have demonstrated considerable phenotypic and genetic heterogeneity within *T. maritimum* using biochemical characterization, serological typing, and molecular fingerprinting approaches such as RAPD-PCR and REP-PCR [20,21,22]. More recently, comparative genomic analyses have confirmed substantial intraspecific diversity, revealing variability in genomic islands, secretion systems, iron acquisition mechanisms, and surface-associated proteins involved in host interactions [23]. Notably, differences in the organization and composition of the O-antigen biosynthesis gene cluster further support ongoing genetic diversification, potentially driven by horizontal gene transfer and host adaptation processes.

Although extensive international studies have documented the phenotypic, serological, and genomic diversity of *T. maritimum* south American salmon aquaculture remains underrepresented. In Chile, existing reports have largely focused on species-level identification or isolated genomic data [12,13,14], leaving the genetics structure, serotype composition, and temporal dynamics of currently circulating strains poorly characterized.

*T. maritimum* diversity was initially documented through phenotypic, biochemical, and serological analyses and later supported by RAPD and REP-PCR fingerprints [20,21], which revealed substantial genetic heterogeneity among isolates. More recent genomics studies have identified the O-antigen biosynthesis gene cluster as a major hotspot of diversification underlying serotype differentiation [22].

Despite the growing body of taxonomic and genomic information available for *T. maritimum* important knowledge gap remains regarding the intraspecific diversity of strains currently circulating in Chilean salmon aquaculture. Temporal shifts in serotype distribution, genetic fingerprint patterns, and virulence variability among recent outbreak isolates from Chilean salmonids have not been systematically evaluated.

Filling these gaps is epidemiologically relevant, as variation within the O-antigen gene cluster directly influences lipopolysaccharide (LPS) structure, a major surface antigen involved in host immune recognition and serotype differentiation. Antigenic variation in LPS has been widely associated with immune evasion, strain replacement, and vaccine mismatch phenomena in aquaculture systems [23,24]. Similarly, strain-level genomic heterogeneity, including differences in virulence-associated loci and secretion systems, has been linked to variability in pathogenicity and outbreaks dynamics in salmonids production systems [3,25]. Such diversity may directly impact the effectiveness of disease control measures, particularly in contexts where autogenous vaccines are formulated based in locally circulating isolates [10,24].

Furthermore, the coexistence of multiple *T. maritimum* serotypes and genetic lineages, together with the broad host range of this pathogen, suggests the potential for interspecies transmission [14,21,23]. However, the genetic relationships and virulence profiles of isolates recovered from different host species in Chile remail poorly understood, limiting our ability to interpret outbreak dynamics at the population level.

In this context, experimental infections assays represent a critical approach to directly assess virulence differences among genetically and serologically distinct *T. maritimum* isolates, providing essential information to support evidence-based surveillance, outbreaks interpretation, and disease control strategies in Chilean salmon aquaculture [25,26,27].

The objective of this study was to characterize *T. maritimum* isolates recovered from salmonid outbreaks occurred in Chile between the years 2020 and 2024 through phenotypic and biochemical profiling, molecular serotyping based on the O-antigen gene cluster (O-AGC), REP-PCR-based genetic fingerprinting, and experimental bath-challenge assays in Atlantic salmon.

## 2. Materials and Methods

### 2.1. Bacterial Strains and Growth Conditions

The 40 Chilean *T. maritimum* isolates used in this study were obtained by Farmacología en Aquacultura Veterinaria FAV S.A. from fish farms of red cusk-eel (*G. chilensis*) (n = 1), rainbow trout (*Oncorhynchus mykiss*) (n = 2), and Atlantic salmon (*S. salar*) (n = 37) during tenacibaculosis outbreaks that occurred in Chile between 2020 and 2024 (Table 1). Reference type strain *T. maritimum* NCIMB 2154^T^ (serotype 01) from red sea bream fingerling *(Pagrus major*) was used as a positive control and for comparative purposes. All isolates were routinely grown on *Flexibacter maritimus* medium (FMM); ref. [28] agar plates or in Marine broth 2216 (BD Difco™, Franklin Lakes, NJ, USA) and aerobically incubated at 20 °C for 72 h. Stock cultures were maintained and stored at −80 °C in Cryobank^®^ cryogenic tubes (Mast Group, Bootle, UK) and Marine broth 2216 (BD Difco™, Franklin Lakes, NJ, USA) supplemented with 10% (*v*/*v*) glycerol.

### 2.2. PCR Identification

The confirmation of *T. maritimum* species for each isolate was carried out by species-specific PCR analysis according to [29] using the set of primers MAR1 and MAR2. Isolation of DNA from each isolate and strain was performed using InstaGene™ Matrix (Bio-Rad, Hercules, CA, USA) following the manufacturer’s instructions. PCR reactions were conducted using the GoTaq^®^ Green Master Mix (Promega, Madison, WI, USA), and an amplification product of 1088 bp compatible with that obtained for the NCIMB 2154^T^-type strain was considered positive for *T. maritimum*. Negative controls consisted of the same reaction mixture but with sterile distilled water instead of template DNA.

### 2.3. Biochemical and Phenotypic Characterization

The phenotypic characterization of the isolates was carried out according to the minimum standards proposed by [30,31]. Isolates were grown on FMM agar plates and incubated at 20 °C for at least 72 h for further evaluations, including colony morphology (shape and color). Gram staining (Merck, Darmstadt, Germany) was tested according to the manufacturer’s instructions. Catalase activity (3% H_2_O_2_) (Merck, Darmstadt, Germany) and cytochrome activity (oxidase) (Merck, Darmstadt, Germany) were determined as described by [32]. The presence of flexirubin-type pigments (20% KOH) (Merck, Darmstadt, Germany) was determined as described by [33]. Sea water tolerance was tested in FMM broth containing 10, 20, 30, 40, 50, 60, 70, 80, 90, and 100% (*v*/*v*) strength seawater. Hydrolysis of elastin (0.5%) (Sigma Aldrich, St. Louis, MO, USA), Tween 80 (1%) (Sigma Aldrich, St. Louis, MO, USA), Tween 20 (1%) (Sigma Aldrich, St. Louis, MO, USA), starch (1%) (Sigma Aldrich, St. Louis, MO, USA), gelatin (1%) (Merck, Darmstadt, Germany), and lecithin (5% sterile egg-yolk suspension) were tested in FMM agar plates with inoculum adjusted at DO_600nm_ 0.1 ± 0.01 for 3–14 days at 20 °C.

### 2.4. Genetic and Antigenic Typing Characterization

The REP-PCR amplifications were performed, according to [21] with some modifications, using Ready-to-Go PCR analysis beads (Amersham Biosciences, Little Chalfont, UK). For REP-PCR, the following 18-mer primers (IDT DNA, Coralville, IA, USA) were utilized: REP 1D (5′-NNN RCGYCG NCA TCM GGC-3′) and REP 2D (5′-RCG YCT TAT CMG GCC TAC-3′), where M is A or C, R is A or G, Y is C or T, and N is any nucleotide [34]. The DNA amplification was performed using Illustra PuReTaq Ready-To-Go PCR Beads (Cytiva, Marlborough, MA, USA). Each PuReTaq bead contains stabilizers, BSA, dATP, dCTP, dGTP, dTTP, ~2.5 units of PuReTaq DNA polymerase, and reaction buffer. The bead was reconstituted to a 25 μL final volume, with a 200 μM concentration of each dNTP in 10 mM Tris-HCl (pH 9.0 at room temperature), 50 mM KCl, 1.5 mM MgCl_2_, and 1 µL of DNA (10 ng/µL). Amplifications were performed in a SureCycler 8800 thermocycler (Agilent Technologies, Santa Clara, CA, USA) programmed as follows: an initial denaturation step at 95 °C for 5 min followed by 35 cycles of denaturation (95 °C for 30 s), annealing (45 °C for 1 min), and extension (65 °C for 5 min), with a final extension step at 70 °C for 10 min. A positive control, consisting of the same reaction mixture but with template DNA extracted from *T. maritimum* reference strain NCIMB 2154^T^, and negative control, consisting of the same reaction mixture but with molecular biology-grade water instead of template DNA, were included in each run.

All isolates were typed using the multiplex PCR-based serotyping scheme described by [22]. mPCR analyses to differentiate type 1-0; type 1-1, type 2-0, type 2-1; type 3-0; type 3-1; type 3-2; and type 4-0 were performed using GoTaq^®^ Green Master Mix (Promega, Madison, WI, USA) and 10 µM of each primer. Positive controls, consisting of the same reaction mixture but with template DNA extracted from *T. maritimum* reference strain NCIMB 2154^T^ (type 1-0), Tm-035 (type 3-1), and Tm-113 (type 4-0) from [13,14] were included. A negative control, consisting of the same reaction mixture but with molecular biology-grade water instead of template DNA, was included in each run.

All PCR products were separated by electrophoresis on a 1.5% agarose gel and stained with SYBR™ Gold (Invitrogen, Carlsbad, CA, USA). The gels were photographed under UV light with a 1 kb ladder (MaestroGen, Inc, Hsinchu, Taiwan) used as a molecular mass marker. To determine a significant difference in the patterns, the reproducibility of results was assessed by repetition of at least three independent REP-PCR assays from three DNA extraction for each sample.

### 2.5. Virulence in Fish

The virulence potential of 19 of the 40 *T. maritimum* isolates recovered from *S. salar* and *G. chilensis* (Tm-222) was assessed according to the molecular serotype and REP-PCR profiling. Experimental challenges were performed using Atlantic salmon weighing approximately 150 g with no prior disease history, sourced from a commercial aquaculture facility in the Los Lagos region, Chile. Prior to transportation, fish were certified free of major Chilean freshwater pathogens (including *Yersinia ruckeri*, *Renibacterium salmoninarum*, *F. psychrophilum*, and infectious pancreatic necrosis virus, among others) by PCR analysis.

Upon arrival at the experimental facility, a total of 450 fish were randomly distributed into fiberglass resin tank (300 L; 20 fish per tank) and acclimatized for 14 days under continuous flow of fresh seawater at 15 ± 1 °C with constant aeration. Fish were fed daily with a commercial pellet diet at 1.5% body weight daily.

Challenges were performed as a single experiment per isolate, using one group of 20 fish, rather than duplicate groups, to comply with animal welfare principles and to maintain an ethically justified number of animals. This experimental design was considered sufficient, as the objective was solely to confirm the virulence potential of each *T. maritimum* isolate.

For each isolate, a single challenge dose was evaluated. Inocula were prepared from overnight Marine Broth cultures (BD Difco™, Franklin Lakes, NJ, USA) incubated at16 °C with agitation (160 rpm). Fish were fasted for 24 h prior to challenge and subsequently exposed by static bath immersion for 18 h, a route of infection that has also been employed in other geographic regions for this pathogen [2,24,25,26,33], using doses ranging from 1.12 × 10^6^ to 1.00 × 10^7^ cells/fish depending on the isolate.

Environmental parameters were maintained throughout the 21-day observation period, with temperature at 15 ± 1 °C, salinity 32 ± 1‰, dissolved oxygen maintained at 90–100% saturation, and tank water was changed twice daily to remove fecal material.

Mortality was monitored daily. Dead fish were removed daily from each tank and subjected to a bacteriological examination by direct streaking of samples (i.e., skin lesion, spleen, and kidney) onto marine agar (BD Difco™, Franklin Lakes, NJ, USA) plates, followed by incubation at 20 °C for 3–4 days. Presumptive *T. maritimum* colonies were confirmed by biochemical characterization and PCR analyses.

**Figure 1:**
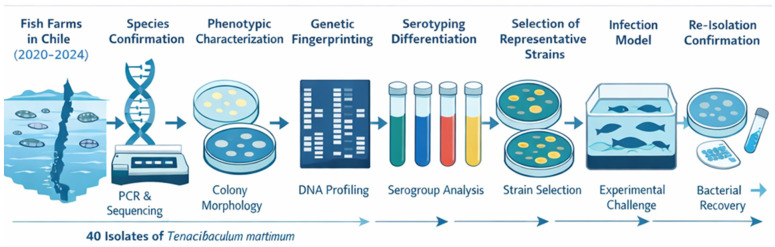
Illustration showing the overview of the study workflow from isolate source to genetic and phenotypic characterization, experimental infection, and integration of genotype-serotype-virulence data (Illustration created with Sora 2).

The analyses and experimental procedures described throughout this study are summarized in Figure 1, which provides an overview of the complete workflow, including isolate collection, phenotypic and molecular characterization, serotyping, strain selection, experimental challenge, and bacteriological confirmation. All animal procedures were conducted in accordance with the Canadian Council on Animal Care Guidelines on the Care and Use of Fish in Research, Teaching, and Testing (https://ccac.ca/Documents/Standards/Guidelines/Fish.pdf (accessed on 20 February 2026)). Ethical approval was granted by the FAV institutional Animal Ethics review committee, under the project titled “Evaluation of a bivalent vaccine against tenacibaculosis caused by *T. maritimum* and *T. dicentrarchi* in the farming of Atlantic salmon (*Salmo salar*) in Chile” (approval code UE-024-005, 12 March 2024).

## 3. Results

### 3.1. Biochemical and Phenotypic Characterization

After isolation and confirmation of purity of the 40 isolates by Gram staining and in vivo observation, confirmatory PCR was performed using the primers described by Toyama et al. (1996) [29], and all isolates amplified the expected 1088 bp product corresponding to *T. maritimum* (Table 1).

Phenotypic analyses show that *T. maritimum* isolates correspond to filamentous Gram-negative bacteria that produce catalase and cytochrome oxidase; however, they lack flexirubin-type pigments. Colonies on FMM agar are flat-shaped, with irregular edges and a pale-yellow color and adhered strongly to the media [21,35,36].

Regarding seawater tolerance, ninety-five percent of the isolates (95%; n = 38) achieved optimal growth at 40–50% seawater supplementation, while isolates Tm-149 and Tm-183 required ≥50% seawater, and twenty-four percent of the isolates (24%; n = 10) grew at 30% seawater. All isolates hydrolyzed Tween 80 and lecithin, while degradation of Tween 20 occurred in 73% of isolates. Gelatin hydrolysis was detected in 21 isolates (52.5%), and starch hydrolysis occurred in fifteen percent (15% n = 6), all belonging to serotype 4-0. No isolates degraded elastin.

### 3.2. Genetic and Antigenic Typing Characterization

**Figure 2:**
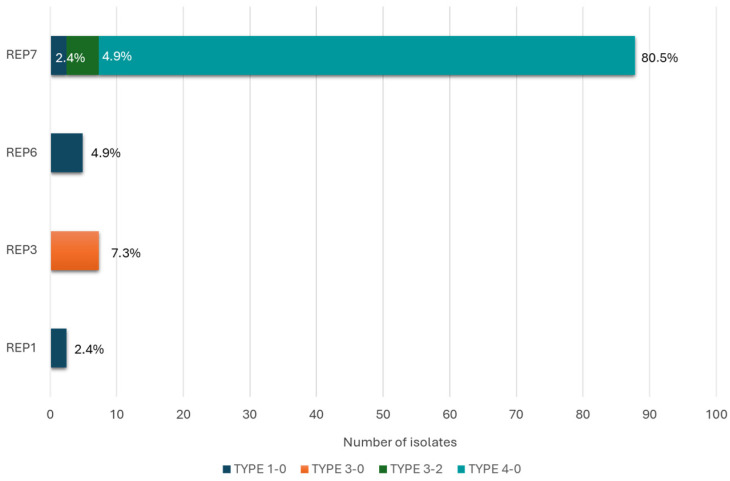
Percentage distribution of the isolates of *T. maritimum* used in this study according to their molecular serotype and REP profile. REP7 presents three serotypes: 4-0, 3-2 and 1-0; REP6 presents only serotype 1-0; REP3 presents only serotype 3-0; and REP corresponds to the reference-type strain NIMB 2154^T^, used as a control.

Multiplex PCR-based serotyping identified four O-AGC serotypes among Chilean isolates as follows: 4-0 (n = 32), 1-0 (n = 3), 3-1 (n = 3), and 3-2 (n = 2). Serotypes 1-0 and 3-2 were detected for the first time in Chile (Table 2, Figure 2).

**Figure 3:**
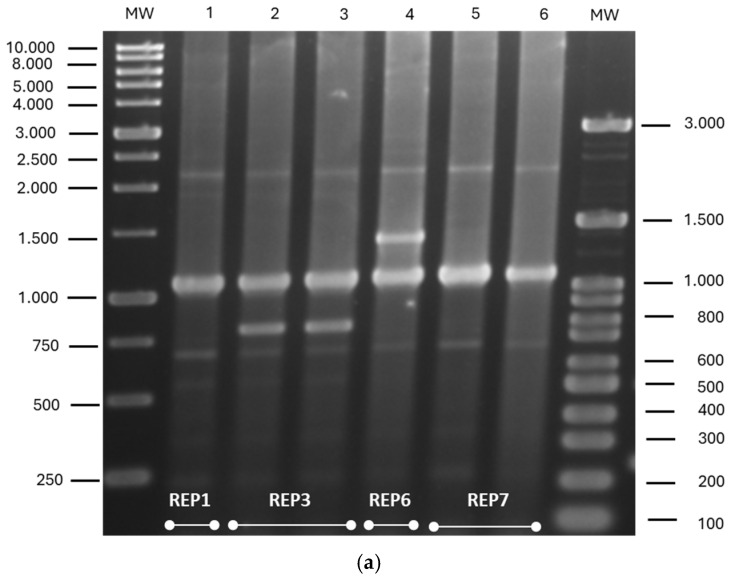

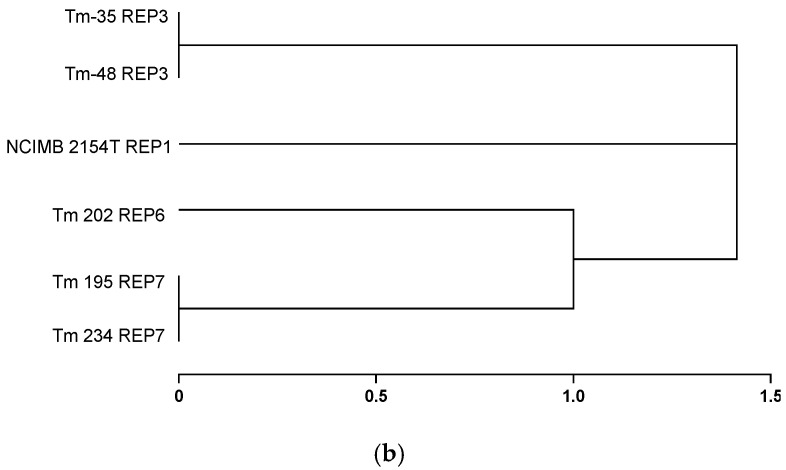
(**a**) Representative REP profiles yielded by the analysis of the isolates of *T. maritimum* used in this study. Molecular weight (MW): AccuRuler 1Kb DNA Ladder (Maestrogen) and AccuRuler 100 bp Plus DNA Ladder. Lines (1): *T. maritimum* NCIMB 2154^T^, (2): Tm-035, (3): Tm-048, (4): Tm-202, (5) Tm-195, and (6): Tm-234. The lines contained 10 µL of PCR reaction. Numbers on the left and right indicate the position of molecular size marker in kb. (**b**) Hierarchical clustering (dendrogram) of REP-PCR profiles showing the genetic relationships among isolates and their grouping into distinct REP types (see Supplementary Material for the original image of Figure 3a).

REP-PCR analysis revealed three isolates matching REP3 patterns previously described. Two novel patterns (REP6 and REP7) were identified. REP6 included isolates Tm-194 and Tm-202 (both type 1-0), whereas REP7 grouped 35 isolates belonging mainly to type 4-0, but also to serotypes 1-0 and 3-2 (Table 2, Figure 3 and Figure S1).

### 3.3. Virulence in Fish

Virulence testing was performed on 19 isolates (Table 3). Serotype 3-1 isolates (Tm-035, Tm-036, Tm-048) caused 50–100% mortality at 21 dpi. Serotype 3-2 isolates showed mortality of 25% (Tm-176) and 85% (Tm-198).

Among the 12 isolates of serotype 4-0 tested, mortality ranged from 0% to 70%. Type 1-0 isolates displayed variable virulence, as follows: Tm-202 reached 100% mortality, Tm-195 reached 65%, and the reference strain NCIMB 2154^T^ produced 50% mortality. Isolate Tm-222 from *G. chilensis* caused 20% mortality in Atlantic salmon.

## 4. Discussion

Biochemical characterization showed an overall homogeneous profile consistent with previous descriptions of *T. maritimum* [21,35]. However, differences in specific hydrolytic activities revealed phenotypic heterogeneity among Chilean isolates. While all strains metabolized Tween 80 and lecithin similarly to the type strain NCIMB 2154^T^, only 52.5% degraded gelatin. This contrasts with earlier reports describing uniform gelatinase activity [35,36], suggesting either temporal variation or strain diversification in local populations. Most non-gelatinolytic isolates belonged to serotype 4-0, indicating that phenotypic variability may occur within dominant antigenic groups. Starch degradation was restricted to a small subset of 4-0 isolates, further supporting intraserotype heterogeneity. Elastin hydrolysis was not detected, in agreement with previous observations [37].

Growth differences under varying salinity conditions indicate ecological flexibility. Although most isolates grew optimally at 50–60% seawater, some tolerated lower salinity (30%), while others required higher salinity, consistent with adaptation to diverse aquaculture environments. These findings suggest that Chilean *T. maritimum* populations are not phenotypically uniform despite sharing core biochemical traits.

O-AGC typing revealed four molecular serotypes (1-0, 3-1, 3-2, and 4-0), with 4-0 predominating (80%). Previous Chilean studies reported serotype 3-1 as dominant [14]; thus, the current predominance of 4-0 may reflect temporal shifts in circulating lineages or broader sampling coverage. The detection of serotypes 1-0 and 3-2 in Atlantic salmon represents an epidemiologically relevant finding, as these had not been previously reported in Chile. Rather than indicating emergence per se, their identification may reflect improved resolution enabled by the O-AGC mPCR scheme [22]. Importantly, isolates within these serotypes displayed variable virulence, indicating that antigenic classification alone does not predict pathogenic potential. Given that the O-antigen constitutes a major immunogenic component of the bacterial surface, the coexistence of multiple serotypes within production systems has practical implications for vaccine formulation. In contexts where autogenous vaccines are implemented, inclusion of locally dominant and virulent serotypes becomes critical to ensure adequate antigenic coverage.

REP-PCR analysis demonstrated substantial genetic heterogeneity, consistent with its continued use as a rapid and discriminatory genotyping tool for strain-level differentiation in epidemiological studies, particularly when whole-genome sequencing is not yet available for all isolates [38,39]. Two previously undescribed amplification patterns (REP6 and REP7) were identified, expanding the known diversity of Chilean isolates. REP7 included most isolates across multiple serotypes, whereas REP6 grouped two 1-0 isolates, including one closely related to the reference strain. The lack of strict concordance between REP profile and O-AGC serotype supports growing genomic evidence that serotype does not necessarily reflect phylogenetic relatedness, as antigenic loci are often subject to horizontal gene transfer and recombination [40,41,42]. This dissociation indicates that serotype distribution alone is insufficient to resolve epidemiological relationships and reinforces the value of integrating molecular serotyping with higher-resolution genotyping approaches for outbreak investigation and surveillance [39,43,44].

Marked heterogeneity in virulence was observed, with cumulative mortality ranging from 0% to 100%. Serotype 3-1 isolates showed consistently high mortality, aligning with previous reports associating certain lineages with acute outbreaks [25,27]. However, high virulence was not restricted to a single serotype, as demonstrated by isolate Tm-202 (1-0), which induced 100% mortality. Serotype 4-0, although predominant, exhibited wide virulence variation (0–70%), indicating that antigenic dominance does not necessarily correlate with pathogenicity. Similarly, isolates sharing identical enzymatic profiles showed divergent mortality outcomes, suggesting that virulence is multifactorial and not fully explained by serotype or basic phenotypic traits. These findings highlight that pathogenic potential in *T. maritimum* reflects a combination of antigenic, genetic, and possibly regulatory factors.

Overall, this study demonstrates that Chilean *T. maritimum* populations exhibit substantial phenotypic, antigenic, genetic, and virulence diversity. The predominance of serotype 4-0, the detection of previously unreported serotypes, and the identification of novel REP profiles indicate a more complex population structure than previously documented. The lack of strict association between serotype, genetic fingerprint, and virulence underscores the need for integrated molecular surveillance strategies. From a practical standpoint, these results support the use of updated, locally representative isolates in autogenous vaccine formulation and emphasize the importance of continuous epidemiological monitoring in salmon aquaculture systems.

## 5. Conclusions

This study characterizes the phenotypic, antigenic, genetic, and virulence diversity of *Tenacibaculum maritimum* isolates circulating in Chilean salmon aquaculture between 2020 and 2024. Although serotype 4-0 predominated, the detection of serotypes 1-0 and 3-2 for the first time in Chile, together with the identification of novel REP profiles (REP6 and REP7), indicates a more complex population structure than previously reported. The lack of strict concordance between serotype, REP profile, and virulence suggests that antigenic classification alone does not reliably predict pathogenic potential. These findings support the implementation of integrated molecular surveillance strategies and the inclusion of locally circulating and virulent isolates in vaccine formulation for Chilean salmon aquaculture.

## Figures and Tables

**Table 1:** Phenotypical and biochemical properties for the 40 Chilean isolates and one reference strain.

Isolate	Host	Outbreak	Average Weight (kg)	Phase	Year	Country	Region	PCR	Catalase	Oxidase	Flexirrubin Pigments	Growth at Dif. SW %	*Degradation of*					
													Elastin	Gelatin	Tween 20	Tween 80	Lecithin	Starch
NCIMB 2154	*P. major*	Yes	*ni*	*ni*	1977	Japan	Chūgoku	+	+	+	−	40–100	−	+	+	+	+	+
Tm-035	*O. mykiss*	Yes	0.3	Smolt	2020	Chile	X	+	+	*w*	−	40–100	−	+	+	+	+	−
Tm-036	*O. mykiss*	Yes	0.3	Smolt	2020	Chile	X	+	+	*w*	−	40–100	−	+	+	+	+	−
Tm-048	*S. salar*	Yes	5.7	Harvest	2021	Chile	X	+	+	*w*	−	40–100	−	+	+	+	+	−
Tm-113	*S. salar*	No	0.3	Smolt	2022	Chile	XI	+	+	*w*	−	30–100	−	−	+	+	+	−
Tm-123	*S. salar*	Yes	4.7	Adult	2022	Chile	X	+	+	*w*	−	40–100	−	−	+	+	+	−
Tm-124	*S. salar*	Yes	4.7	Adult	2022	Chile	X	+	+	*w*	−	40–100	−	−	−	+	+	−
Tm-125	*S. salar*	Yes	1.8	Adult	2022	Chile	XI	+	+	*w*	−	40–100	−	−	+	+	+	−
Tm-136	*S. salar*	No	1.6	Adult	2022	Chile	X	+	+	+	−	40–100	−	+	+	+	+	−
Tm-145	*S. salar*	*ni*	4.5	Adult	2022	Chile	X	+	+	+	−	40–100	−	+	+	+	+	−
Tm-147	*S. salar*	*ni*	4.5	Adult	2022	Chile	X	+	+	+	−	40–100	−	+	+	+	+	−
Tm-149	*S. salar*	*ni*	5.2	Harvest	2022	Chile	X	+	+	+	−	50–100	−	+	+	+	+	−
Tm-158	*S. salar*	*ni*	*ni*	*ni*	2022	Chile	X	+	+	+	−	40–100	−	+	+	+	+	−
Tm-173	*S. salar*	No	1.7	Adults	2023	Chile	X	+	+	+	−	30–100	−	−	+	+	+	−
Tm-174	*S. salar*	No	1.7	Adults	2023	Chile	X	+	+	+	−	30–100	−	−	+	+	+	−
Tm-176	*S. salar*	No	0.9	Adult	2022	Chile	X	+	+	+	−	30–100	−	−	−	+	+	−
Tm-183	*S. salar*	No	1.7	Adult	2023	Chile	X	+	+	+	−	30–100	−	−	+	+	+	−
Tm-184	*S. salar*	No	1.7	Adult	2023	Chile	X	+	+	+	−	30–100	−	−	+	+	+	−
Tm-191	*S. salar*	Yes	1.0	Adult	2023	Chile	XI	+	+	+	−	30–100	−	−	−	+	+	−
Tm-192	*S. salar*	Yes	4.5	Adult	2023	Chile	XI	+	+	+	−	40–100	−	−	+	+	+	−
Tm-193	*S. salar*	Yes	4.5	Adult	2023	Chile	XI	+	+	+	−	60–100	−	−	−	+	+	−
Tm-194	*S. salar*	Yes	4.9	Adult	2023	Chile	XI	+	+	+	−	40–100	−	−	+	+	+	−
Tm-195	*S. salar*	Yes	1.0	Adults	2023	Chile	XI	+	+	+	−	40–100	−	−	+	+	+	−
Tm-198	*S. salar*	Yes	4.9	Adult	2023	Chile	XI	+	+	+	−	40–100	−	−	−	+	+	−
Tm-200	*S. salar*	Yes	4.9	Adult	2023	Chile	XI	+	+	+	−	30–100	−	+	−	+	+	−
Tm-202	*S. salar*	Yes	3.6	Adult	2023	Chile	X	+	+	+	−	40–100	−	−	+	+	+	−
Tm-208	*S. salar*	Yes	5.0	Adult	2023	Chile	X	+	+	+	−	40–100	−	+	−	+	+	−
Tm-215	*S. salar*	Yes	0.4	Smolt	2023	Chile	X	+	+	+	−	30–100	−	+	−	+	+	+
Tm-218	*S. salar*	Yes	0.4	Smolt	2023	Chile	X	+	+	+	−	30–100	−	+	−	+	+	+
Tm-219	*S. salar*	Yes	5.6	Harvest	2023	Chile	X	+	+	+	−	30–100	−	+	+	+	+	−
Tm-220	*S. salar*	Yes	5.6	Harvest	2023	Chile	X	+	+	+	−	30–100	−	+	+	+	+	−
Tm-222	*G. chilensis*	Yes	0.2	Juvenile	2023	Chile	IV	+	+	+	−	30–100	−	+	+	+	+	+
Tm-225	*S. salar*	Yes	2.4	Adult	2023	Chile	X	+	+	+	−	30–100	−	+	+	+	+	+
Tm-227	*S. salar*	Yes	2.4	Adult	2023	Chile	X	+	+	+	−	30–100	−	+	+	+	+	+
Tm-228	*S. salar*	Yes	4.6	Adult	2023	Chile	X	+	+	+	−	40–100	−	+	+	+	+	+
Tm-232	*S. salar*	Yes	5.6	Harvest	2023	Chile	X	+	+	+	−	40–100	−	+	+	+	+	−
Tm-233	*S. salar*	*ni*	5.6	Harvest	2023	Chile	X	+	+	+	−	40–100	−	+	+	+	+	−
Tm-234	*S. salar*	*ni*	5.6	Harvest	2023	Chile	X	+	+	+	−	40–100	−	−	+	+	+	−
Tm-238	*S. salar*	*ni*	2.3	Adult	2023	Chile	XI	+	+	+	−	40–100	−	+	+	+	+	−
Tm-247	*S. salar*	Yes	4.9	Adult	2024	Chile	XI	+	+	+	−	40–100	−	−	−	+	+	−
Tm-248	*S. salar*	Yes	4.5	Adult	2024	Chile	X	+	+	+	−	40–100	−	–	–	+	+	–

Note: Strains were incubated at 20 °C for 48–72 h. Abbreviations: (−) negative reaction, (+) positive reaction, (*ni*): not informed, (*w*) weak reaction.

**Table 2:** Results of the isolates of *T. maritimum* used in this study by PCR identification of O-AGCs and typing by REP-PCR according to [21].

Isolate	Host	PCR	
		Type	REP
NCIMB 2154	*P. major*	1-0	1
Tm-035	*O. mykiss*	3-1	3
Tm-036	*O. mykiss*	3-1	3
Tm-048	*S. salar*	3-1	3
Tm-113	*S. salar*	4-0	7
Tm-123	*S. salar*	4-0	7
Tm-124	*S. salar*	4-0	7
Tm-125	*S. salar*	4-0	7
Tm-136	*S. salar*	4-0	7
Tm-145	*S. salar*	4-0	7
Tm-147	*S. salar*	4-0	7
Tm-149	*S. salar*	4-0	7
Tm-158	*S. salar*	4-0	7
Tm-176	*S. salar*	3-2	7
Tm-173	*S. salar*	4-0	7
Tm-174	*S. salar*	4-0	7
Tm-183	*S. salar*	4-0	7
Tm-184	*S. salar*	4-0	7
Tm-191	*S. salar*	4-0	7
Tm-192	*S. salar*	4-0	7
Tm-193	*S. salar*	4-0	7
Tm-194	*S. salar*	1-0	6
Tm-195	*S. salar*	1-0	7
Tm-198	*S. salar*	3-2	7
Tm-200	*S. salar*	4-0	7
Tm-202	*S. salar*	1-0	6
Tm-208	*S. salar*	4-0	7
Tm-215	*S. salar*	4-0	7
Tm-218	*S. salar*	4-0	7
Tm-219	*S. salar*	4-0	7
Tm-220	*S. salar*	4-0	7
Tm-222	*G. chilensis*	4-0	7
Tm-225	*S. salar*	4-0	7
Tm-227	*S. salar*	4-0	7
Tm-228	*S. salar*	4-0	7
Tm-232	*S. salar*	4-0	7
Tm-233	*S. salar*	4-0	7
Tm-234	*S. salar*	4-0	7
Tm-238	*S. salar*	4-0	7
Tm-247	*S. salar*	4-0	7
Tm-248	*S. salar*	4-0	7

To ensure reproducibility, PCR assays were conducted independently in triplicate.

**Table 3:** Results of virulence assays at 21 days post-exposure.

Group	Number of Fish	Isolate	O-AGC/REP	Bacterial Bath Concentration (Cell × mL^−1^)	Cumulative Percent Mortality
1	20	NCIMB 2154^T^	1-0/1	5.11 × 10^6^	50%
2	20	Tm-035	3-1/4	4.56 × 10^6^	85%
3	20	Tm-036	3-1/4	5.00 × 10^6^	50%
4	20	Tm-048	3-1/4	5.60 × 10^6^	100%
5	20	Tm-192	4-0/2	4.40 × 10^6^	45%
6	20	Tm-222	4-0/2	5.00 × 10^6^	20%
7	20	Tm-149	4-0/2	5.03 × 10^6^	70%
8	20	Tm-145	4-0/2	5.05 × 10^6^	70%
9	20	Tm-193	4-0/2	4.83 × 10^6^	5%
10	20	Tm-176	3-2/2	1.12 × 10^6^	25%
11	20	Tm-219	4-0/2	5.00 × 10^6^	35%
12	20	Tm-113	4-0/2	7.04 × 10^6^	70%
13	20	Tm-195	1-0/2	1.28 × 10^6^	65%
14	20	Tm-198	3-2/2	5.00 × 10^6^	85%
15	20	Tm-202	1-0/3	5.00 × 10^6^	100%
16	20	Tm-124	4-0/2	8.32 × 10^6^	50%
17	20	Tm-125	4-0/2	8.24 × 10^6^	20%
18	20	Tm-136	4-0/2	1.00 × 10^7^	10%
19	20	Tm-233	4-0/2	1.00 × 10^7^	0%
20	20	Tm-247	4-0/2	1.00 × 10^7^	0%
21	20	Control (MB)	-	500 mL	-

MB (Marine Broth). Cumulative percent mortality of Atlantic salmon bath challenged with *T. maritimum* is shown for each group in the virulence experiments; in general, the mortality curve for each group had a sigmoid shape.

## Data Availability

The data presented in this study are available on request from the corresponding author.

## Supplementary Materials

The following supporting information can be downloaded at: https://www.mdpi.com/article/10.3390/pathogens15070744/s1, Figure S1: Original image of Figure 3a.

## Abbreviations

The following abbreviations are used in this manuscript:

PCR	Polymerase chain reaction
mPCR	Multiplex PCR
REP-PCR	Repetitive extragenic palindromic PCR
RAPD-PCR	Random amplified polymorphic DNA PCR
O-AGC	O-antigen gene cluster
DNA	Deoxyribonucleic acid
bp	Base Pairs
dNTP	Deoxynucleotide triphosphate
BSA	Bovine serum albumin
FMM	Flexibacter maritimus medium
MB	Marine broth
UV	Ultraviolet
KOH	Potassium hydroxide
H_2_O_2_	Hydrogen peroxide
DO600	Optical density at 600 nm
rpm	Revolutions per minute
USA	United States
UK	United Kingdom
dpi	Days post-infection
LPS	Lipopolysaccharide
OMVs	Outer Membrane Vesicles
SERNAPESCA	Servicio Nacional de Pesca y Acuicultura
SUBPESCA	Subsecretaría de Pesca y Acuicultura
ANID	Agencia Nacional de Investigación y Desarrollo
NCIMB	National Collection of Industrial, Marine and Food Bacteria
MW	Molecular weight
