# Supplementary figures and images for: Phenotypic, Genetic, and Virulence Characterization of Tenacibaculum maritimum Isolates Recovered from Salmonid Outbreaks in Chile
Source: Pathogens. 2026 Jul 15;15(7):744. doi: 10.3390/pathogens15070744 (PMC13414609; doi:10.3390/pathogens15070744)

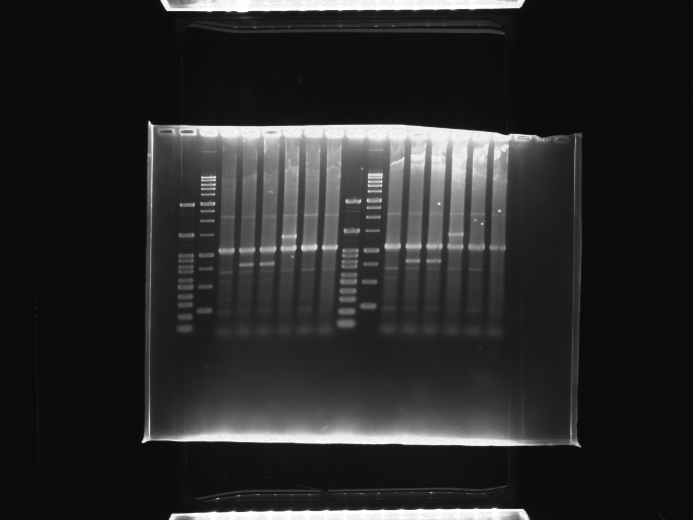

Supplement: Supplementary file 1 [file pathogens-15-00744-s001.zip › Figure S1.tiff]
